# Pharmacological profile and efficiency *in vivo* of diflapolin, the first dual inhibitor of 5-lipoxygenase-activating protein and soluble epoxide hydrolase

**DOI:** 10.1038/s41598-017-09795-w

**Published:** 2017-08-24

**Authors:** Ulrike Garscha, Erik Romp, Simona Pace, Antonietta Rossi, Veronika Temml, Daniela Schuster, Stefanie König, Jana Gerstmeier, Stefanie Liening, Markus Werner, Heiner Atze, Sandra Wittmann, Christina Weinigel, Silke Rummler, Gerhard K. Scriba, Lidia Sautebin, Oliver Werz

**Affiliations:** 1 Chair of Pharmaceutical/Medicinal Chemistry, Institute of Pharmacy, Friedrich-Schiller-University Jena, Philosophenweg 14, D-07743 Jena, Germany; 2 Department of Pharmacy, School of Medicine, University of Naples Federico II, 80131 Naples, Italy; 3 Department of Pharmacy / Pharmaceutical Chemistry and Center for Molecular Biosciences Innsbruck (CMBI), University of Innsbruck, Innrain 80-82, A-6020 Innsbruck, Austria; 4 Institute of Pharmaceutical Chemistry, Goethe University Frankfurt, 60438 Frankfurt, Germany; 5 Institute of Transfusion Medicine, University Hospital Jena, 07743 Jena, Germany

## Abstract

Arachidonic acid (AA) is metabolized to diverse bioactive lipid mediators. Whereas the 5-lipoxygenase-activating protein (FLAP) facilitates AA conversion by 5-lipoxygenase (5-LOX) to pro-inflammatory leukotrienes (LTs), the soluble epoxide hydrolase (sEH) degrades anti-inflammatory epoxyeicosatrienoic acids (EETs). Accordingly, dual FLAP/sEH inhibition might be advantageous drugs for intervention of inflammation. We present the *in vivo* pharmacological profile and efficiency of *N*-[4-(benzothiazol-2-ylmethoxy)-2-methylphenyl]-*N*′-(3,4-dichlorophenyl)urea (diflapolin) that dually targets FLAP and sEH. Diflapolin inhibited 5-LOX product formation in intact human monocytes and neutrophils with IC_50_ = 30 and 170 nM, respectively, and suppressed the activity of isolated sEH (IC_50_ = 20 nM). Characteristic for FLAP inhibitors, diflapolin (I) failed to inhibit isolated 5-LOX, (II) blocked 5-LOX product formation in HEK cells only when 5-LOX/FLAP was co-expressed, (III) lost potency in intact cells when exogenous AA was supplied, and (IV) prevented 5-LOX/FLAP complex assembly in leukocytes. Diflapolin showed target specificity, as other enzymes related to AA metabolism (i.e., COX1/2, 12/15-LOX, LTA_4_H, LTC_4_S, mPGES_1_, and cPLA_2_) were not inhibited. In the zymosan-induced mouse peritonitis model, diflapolin impaired vascular permeability, inhibited cysteinyl-LTs and LTB_4_ formation, and suppressed neutrophil infiltration. Diflapolin is a highly active dual FLAP/sEH inhibitor *in vitro* and *in vivo* with target specificity to treat inflammation-related diseases.

## Introduction

The arachidonic acid (AA) cascade plays a central role in the biosynthesis of lipid mediators (LMs) with pro-inflammatory but also anti-inflammatory properties^1^. In mammalian cells, AA is released from phospholipids by cytosolic phospholipase A_2_ (cPLA_2_) upon stimulation. Liberated AA can be converted via three different pathways to bioactive LMs: cyclooxygenases (COXs) catalyze the initial step in the formation of inflammation-initiating prostaglandins (PGs) and thromboxane (TX), whereas lipoxygenases (LOXs) form hydroperoxyeicosatetra enoic acids (HPETEs), leukotrienes (LTs), and lipoxins (LXs)^2^. Moreover, AA is transformed by cytochrome P450 enzymes to monohydroxyeicosatetraenoic acids (HETEs) and epoxyeicosatrienoic acids (EETs)^3, 4^. EETs possess anti-inflammatory properties and are degraded by soluble epoxide hydrolase (sEH) to the corresponding diols (dihydroxyeicosatrienoic acids (DiHETrEs)) with associated loss of beneficial effects.

COX inhibitors that block PG and TX formation have been intensively studied and are widely used to treat pain and inflammation, albeit with frequent and severe side-effects^5^. Despite significant efforts in developing compounds that interfere with the other pathways of the AA cascade (e.g. 5-LOX), the respective candidates failed in clinical trials due to unpredicted side effects or lack of efficacy^6^. Targeting solely one pathway out of the AA cascade could be one reason for this issue^7^, which is well-known for shunting of AA towards the 5-LOX pathway due to inhibition of PG/TX formation by aspirin or other COX inhibitors^8^. Thus, compounds that selectively act on multiple targets (so-called designed multiple ligands – DML)^9, 10^ may be appropriate to suppress the biosynthesis of pro-inflammatory LMs but maintain anti-inflammatory LMs. Such agents may be advantageous over single-interfering drugs and may represent a promising pharmacological approach for intervention with complex diseases as inflammation^9, 11–14^. We recently discovered ((*N*-[4-(benzothiazol-2-ylmethoxy)-2-methylphenyl]-*N*′-(3,4-dichlorophenyl)urea; compound 5 in ref. 15) as the first dual inhibitor of 5-lipoxygenase-activating protein (FLAP) and sEH by using a pharmacophore-based virtual screening^15^. This compound is now designated diflapolin. FLAP, a nuclear membrane integral protein^16, 17^ assists 5-LOX in LT biosynthesis, and FLAP inhibitors (e.g. MK886, BAY-X 1005) efficiently abolish LT formation *in vitro* and *in vivo* ^18, 19^. sEH degrades EETs with anti-inflammatory and antihypertensive properties to DiHETrEs that are assumed to be pro-inflammatory with additional detrimental properties^20^. Therefore, sEH inhibition is not only elevating EET levels, it rather stabilizes epoxy-fatty acids with favorable actions. It was recently reported that co-administration of a sEH inhibitor with a FLAP inhibitor enhanced the anti-inflammatory activities in a murine model^21^, whereas sole inhibition of sEH lead to albuminuria^22^. These data support the development of dual FLAP/sEH inhibitors to achieve better therapeutic effects due to simultaneous suppression of pro-inflammatory LTs and DiHETrEs but maintaining anti-inflammatory EETs. Here, we present the molecular pharmacological profile and the effectiveness of the first dual FLAP/sEH inhibitor diflapolin^15^ using cell-free and cell-based analysis of biosynthetic pathways of the AA cascade as well as animal models of inflammation. We find that diflapolin is target-specific for sEH and FLAP with strong potencies and represents a highly effective anti-inflammatory compound.

## Results

### Diflapolin inhibits cellular 5-LOX product formation without affecting 5-LOX in cell-free assays

**Figure 1:**
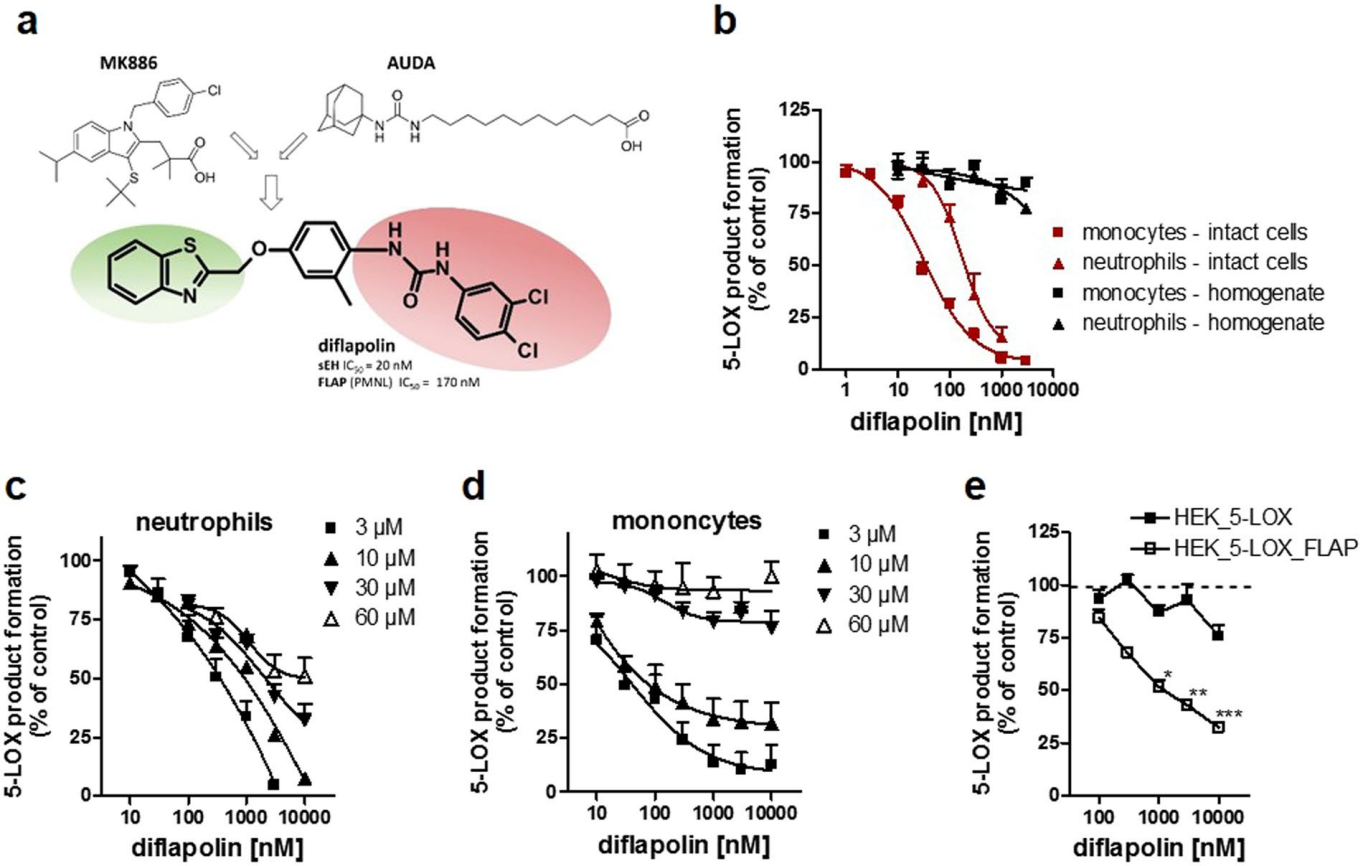
Diflapolin inhibits cellular 5-LOX product formation by targeting FLAP. (**a**) Chemical structure of the dual FLAP/sEH inhibitor diflapolin, with potential pharmacophoric moieties of typical FLAP (MK886) and sEH (AUDA) inhibitors. (**b**) Inhibition of 5-LOX product formation in human monocytes and neutrophils and in corresponding cell homogenates. Cells were pre-incubated with diflapolin (or 0.1% DMSO as vehicle) for 15 min and stimulated with 2.5 µM Ca^2+^-ionophore A23187 for 10 min. Cell homogenates were pre-incubated with diflapolin (or 0.1% DMSO) for 10 min at 4 °C, pre-warmed at 37 °C for 30 sec, and 20 µM AA plus 1 mM CaCl_2_ was added for another 10 min at 37 °C. 5-LOX products were analyzed by HPLC. (**c**,**d**) Effects of exogenous AA on the potency of diflapolin for inhibition of 5-LOX product formation in neutrophils (**c**) and monocytes (**d**). Cells were pre-treated by diflapolin (or 0.1% DMSO as vehicle) at 37 °C for 15 min, and subsequently activated by 2.5 µM Ca^2+^-ionophore A23187 plus the indicated concentrations of exogenous AA for another 10 min. (**e**) HEK293 cells expressing 5-LOX or 5-LOX and FLAP were pre-incubated with diflapolin and stimulated with 5 µM Ca^2+^-ionophore A23187 plus 3 µM AA for 10 min at 37 °C. 5-LOX products were analyzed by HPLC. Data, expressed as percentage of vehicle control (=100%), are given as means ± S.E.M, n = 3 *p < 0.05; **p < 0.005; ***p < 0.001 *vs*. vehicle control (ANOVA + Bonferroni with logarithmized values).

Based on a pharmacophore-based virtual screening campaign, diflapolin was identified as most potent agent out of 20 hit compounds that dually inhibited FLAP and sEH in simple screening assays^15^. The structure of diflapolin is composed of a urea moiety (present in the sEH reference inhibitor AUDA) potentially binding to sEH as a mimetic of epoxides, and an aromatic heterocyclic scaffold (benzothiazole, seemingly reflecting the indole scaffold of the reference FLAP inhibitor MK886) that may primarily confer FLAP interference (Fig. 1a). We first aimed to investigate the interference of diflapolin with FLAP and thus with 5-LOX product biosynthesis in more detail. Since FLAP does apparently not possess any measurable enzyme activity that can be readily monitored in a cell-free assay, functional interference of a given compound with FLAP requires indirect analysis of 5-LOX activation and product formation in intact cells^23^. In intact monocytes and neutrophils from human peripheral blood stimulated with Ca^2+^-ionophore, diflapolin effectively inhibited the formation of LTB_4_ and its isomers and of 5-H(p)ETE with IC_50_ values of 30 and 170 nM, respectively (Fig. 1b). In order to exclude direct inhibition of 5-LOX, diflapolin was analyzed against 5-LOX activity in cell-free assays. Diflapolin, up to 10 µM, did not significantly inhibit the activity of isolated human recombinant 5-LOX (not shown) or of 5-LOX in homogenates of neutrophils and monocytes (Fig. 1b). The same pattern of interference with 5-LOX product formation was observed for the FLAP inhibitor MK886 (IC_50_ monocytes: ~3 nM, neutrophils: 10–14 nM; IC_50_ 5-LOX in cell-free assays: >10 µM, not shown), which is in agreement with the literature^24^. In contrast, the direct 5-LOX inhibitor zileuton inhibited 5-LOX activity in the cell-based (monocytes, neutrophils) and cell-free assays about equally well (IC_50_ = 1.5 and 0.8 µM, respectively), as reported^25^. A typical feature of FLAP inhibitors is their loss of efficiency, when cells are stimulated for 5-LOX product formation in the presence of exogenous AA, since (I) FLAP inhibitors compete with AA binding within the active site of FLAP^26^, and (II) ample AA supply may circumvent the requirement of FLAP for cellular 5-LOX product formation^27, 28^. In both monocytes and neutrophils, increasing levels of AA (up to 60 µM) sequentially reduced the inhibitory potency of diflapolin and shifted the IC_50_ values from 30 and 170 nM, respectively, (no AA supplementation) to >10 µM (at 60 µM AA) (Fig. 1c/d). These data suggest that diflapolin binds in the fatty acid substrate (AA) pocket of FLAP.

To confirm the hypothesis that diflapolin acts as FLAP inhibitor, we studied suppression of 5-LOX product formation in stably transfected HEK293 cells that either express 5-LOX plus FLAP or 5-LOX alone. Note that induction of 5-LOX product formation in HEK293 cells requires supplementation of exogenous AA (regardless of FLAP)^29^ and we therefore stimulated the cells with A23187 plus 3 µM AA. Diflapolin strongly inhibited 5-LOX product formation in intact HEK293 cells expressing both 5-LOX and FLAP, whereas in HEK293 cells deficient in FLAP, 5-LOX product formation was hardly impaired by diflapolin (Fig. 1e). Together, these data show that diflapolin inhibits 5-LOX product formation only when FLAP is operative supporting FLAP as target of diflapolin.

### Diflapolin inhibits epoxide hydrolase activity of sEH without affecting the phosphatase activity

**Figure 2:**
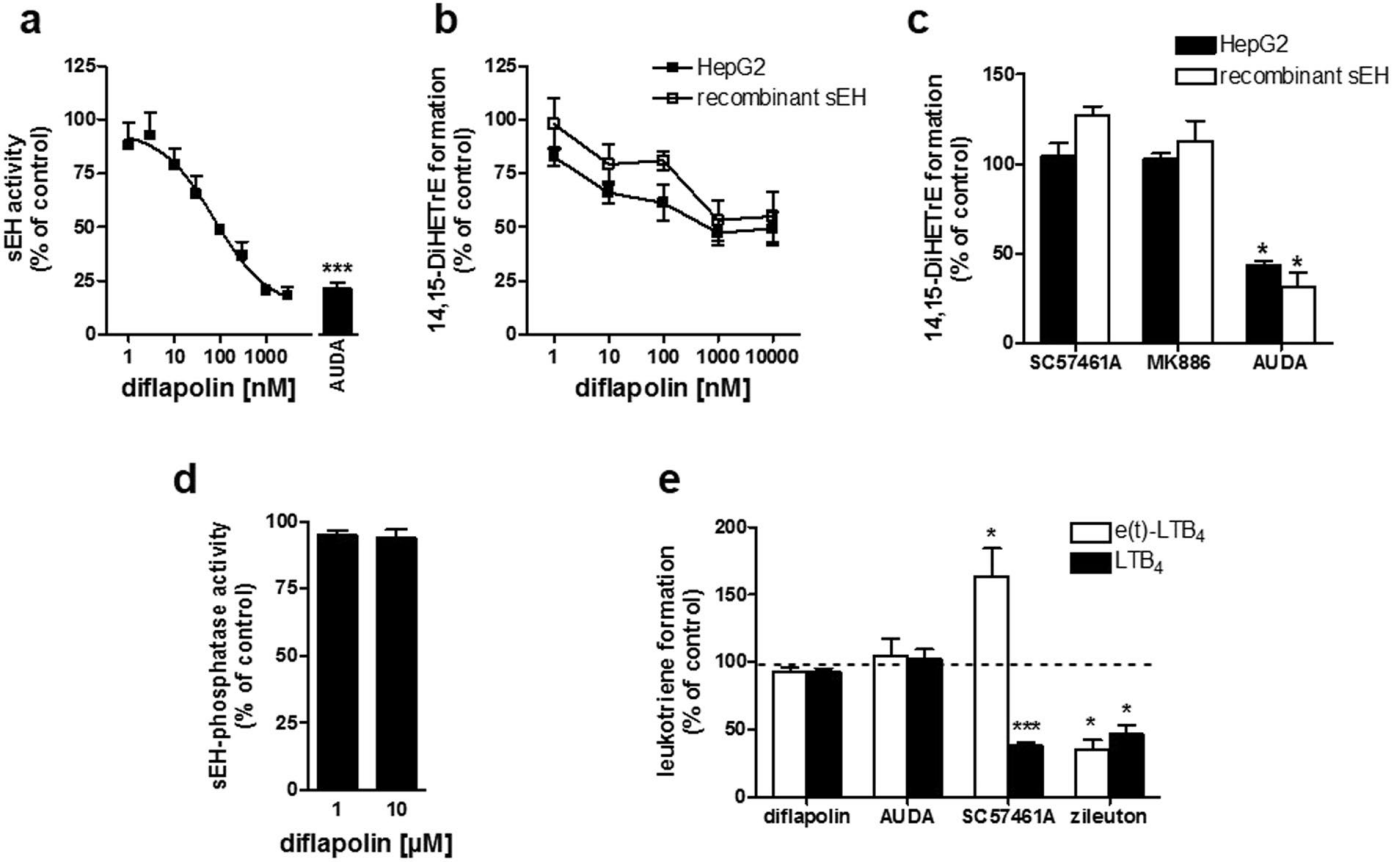
Diflapolin inhibits epoxide hydrolase activity of sEH. (**a**) The epoxide hydrolase activity of human recombinant sEH was analyzed in fluorescence-based cell-free assay. Diflapolin, AUDA (300 nM), or DMSO (vehicle, 0.1%) was added to sEH and after 10 min at 4 °C, the reaction was started by addition of the substrate (50 µM PHOME) and stopped after 60 min before analyzing the fluorescent product. Data are expressed in percentage of control and are given as means ± S.E.M., n = 3–4, ***p < 0.001; *vs* vehicle (paired t-test). (**b**/**c**) sEH activity was analyzed in intact HepG2 cells and for recombinant sEH. Cells and enzyme were pre-incubated with DMSO (vehicle, 0.1%), diflapolin (indicated concentrations), SC57464A (0.3 µM), AUDA (5 µM) or MK886 (0.3 µM), and then incubated with 14,15-EET. Amounts of 14,15-EET and 14,15-DiHETrE were analyzed by UPLC-MS/MS. Data are expressed as percentage of control and are given as means ± S.E.M., n = 3, *p < 0.05; versus vehicle (paired t-test). (**d**) Phosphatase activity of sEH was analyzed in a fluorescence-based cell-free assay. Diflapolin or DMSO (vehicle, 0.1%) was added to human recombinant phosphatase domain of sEH for 10 min at 4 °C, the reaction was initiated by addition of DiFMUP (300 µM) and fluorescence was analyzed for 45 min. Data are expressed as percentage of vehicle control (100%), are given as means ± S.E.M., n = 3 *p < 0.05 *vs*. vehicle control (paired t-test). (**e**) Co-incubations of human recombinant 5-LOX and LTA_4_-H in PBS plus 1 mM EDTA pre-incubated with diflapolin (10 µM), AUDA (10 µM), SC57461A (0.3 µM), zileuton (3 µM), or vehicle (0.1% DMSO) for 10 min on ice and subsequently stimulated by 20 µM AA and CaCl_2_ for 10 min at 37 °C. LTB_4_ isomers were analyzed by HPLC. Data are expressed as percentage of vehicle control (100%), are given as means ± S.E.M., n = 3–4. *p < 0.05 *vs*. vehicle control (paired t-test with logarithmized values).

sEH is a bifunctional enzyme with a C-terminal epoxide hydrolase (EH) and an N-terminal phosphatase activity that operate independent from each other^30^. In a cell-free assay, diflapolin reduced the EH activity of human recombinant sEH with an IC_50_ of 20 nM (Fig. 2a), comparable to the activity of AUDA (IC_50_ = 69 nM), a well-recognized reference inhibitor of sEH^31^. sEH is constitutively expressed in the human liver cancer cell line HepG2 making it suitable as cell-based test system for evaluation of diflapolin for sEH inhibition in the cellular context. HepG2 cells were pre-treated with diflapolin and control inhibitors, and incubated with the sEH substrate 14,15-EET. sEH activity was then analyzed by monitoring 14,15-DiHETrE formation using UPLC-MS/MS. Diflapolin as well as AUDA inhibited cellular sEH activity to ~50% at 1 µM (Fig. 2b). Further decrease at concentrations up to 10 µM could not be observed, probably due to EET degradation that was sEH-independent and potentially non-enzymatic, as recombinant sEH in the corresponding cell-free assay yielded comparable results (Fig. 2b). AUDA reduced the sEH activity in a comparable manner, whereas MK886 and SC57461A (LTA_4_-H inhibitor) had no impact on sEH in the cell-based sEH assay (Fig. 2c).

Next, we tested diflapolin against the phosphatase activity of sEH in a cell-free assay. Of interest, diflapolin failed to inhibit the phosphatase activity even at high concentrations (10 µM) (Fig. 2d). In order to demonstrate that diflapolin as a specific inhibitor of the hydrolase activity of sEH, its effect on LTA_4_-H was determined. LTA_4_-H hydrolyses the epoxide in LTA_4_ that is produced from AA by 5-LOX in a co-incubation experiment using isolated 5-LOX and LTA_4_-H, where LTB_4_ is formed. Diflapolin failed to inhibit LTA_4_-H activity (i.e. LTB_4_ formation) up to 10 µM, compared to the LTA_4_-H inhibitor SC57461A (IC_50_ of 0.1 µM against recombinant LTA_4_-H)^32^ that blocked LTB_4_ biosynthesis and shifted the conversion of LTA_4_ towards the non-enzymatically formed trans-isomers of LTB_4_ (Fig. 2e). AUDA (10 µM) showed the same pattern as diflapolin, whereas zileuton (3 µM) as a direct 5-LOX inhibitor reduced the formation of all LTB_4_ isomers (Fig. 2e), as expected due to reduced LTA_4_ formation.

### Effects of diflapolin on other eicosanoid biosynthetic enzymes

**Figure 3:**
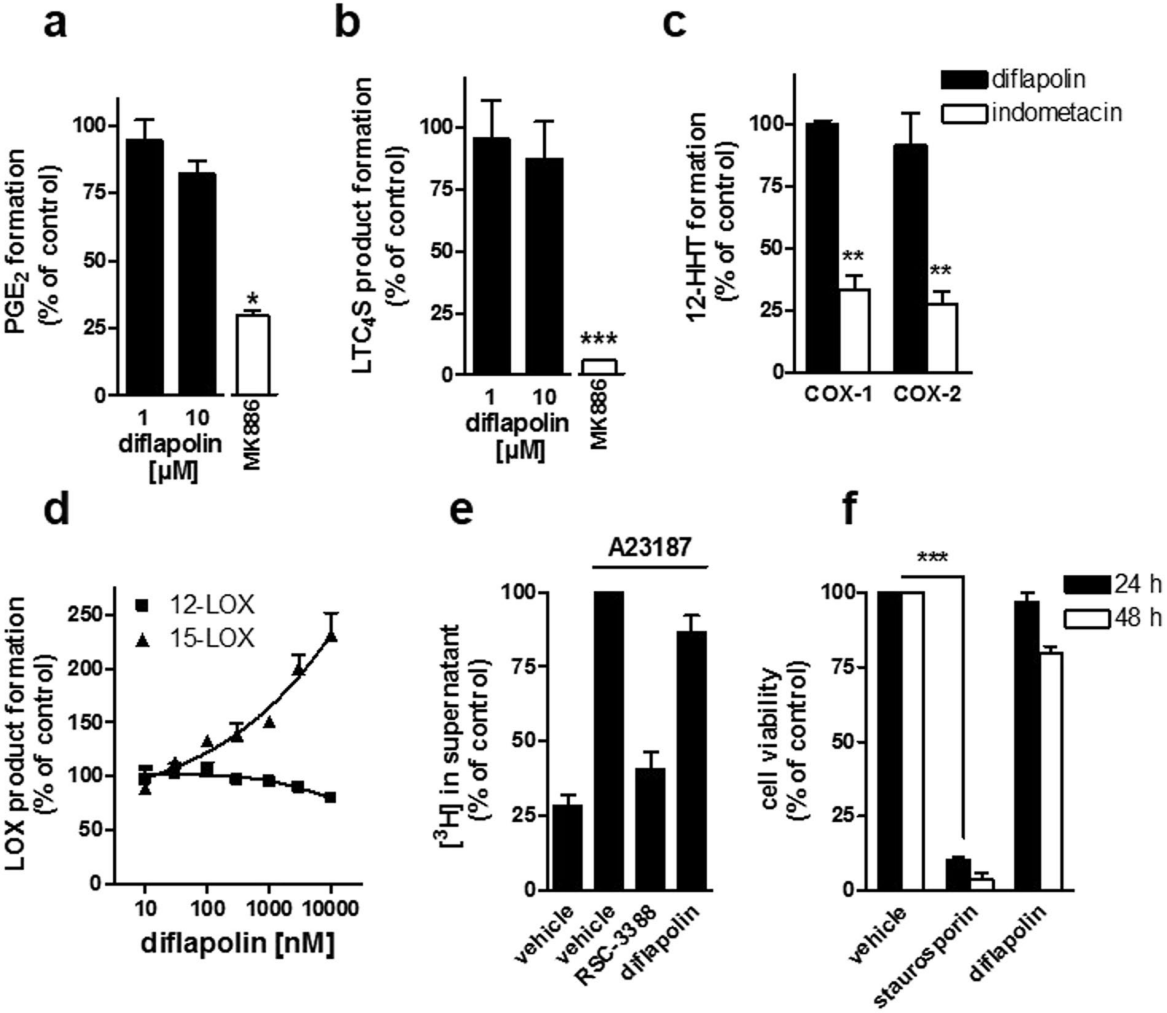
Diflapolin shows target specificity within the AA cascade without cytotoxicity. (**a**) mPGES-1 activity. Microsomes of IL-1β-stimulated A549 cells were pretreated with diflapolin, 10 µM MK886, or vehicle for 10 min on ice and stimulated with 20 µM PGH_2_. After 1 min at 4 °C, PGE_2_ formation was analyzed by HPLC. (**b**) LTC_4_S activity. Microsomes of LTC_4_S-expressing HEK293 cells were pretreated with diflapolin, MK886 (10 µM), or vehicle for 10 min on ice with subsequent addition of LTA_4_-methyl ester. After 10 min at 4 °C, LTC_4_-methyl ester was analyzed by UPLC-MS/MS. (**c**) COX-1/2 activity. In cell-free assays, purified ovine COX-1 and recombinant human COX-2 were pretreated with diflapolin (10 µM), indometacin (10 µM) or vehicle (0.1% DMSO) for 5 min on ice and stimulated with AA (5 and 2 µM for COX-1 and -2, respectively for 10 min at 37 °C. 12-HHT was analyzed by HPLC. (**d**) Effect of diflapolin on 12- and 15-LOX. Intact neutrophils were pre-incubated by diflapolin or vehicle (0.1% DMSO) and stimulated with 2.5 µM Ca^2+^-ionophore plus 20 µM AA for 10 min at 37 °C. 12- and 15-HETE were determined by HPLC. (**e**) AA release. [^3^H]AA-labeled neutrophils were pretreated with diflapolin (1 µM), RSC-3388 (10 µM) or vehicle (0.1% DMSO) and stimulated by 2.5 µM Ca^2+^-ionophore for 15 min. Radioactivity of the supernatant was analyzed by scintillation counting. (**f**) Cell viability assay. Monocytes were treated with diflapolin (10 µM), staurosporin (3 µM) or vehicle (0.3% DMSO) for 24 and 48 h, respectively. Cell viability was determined by MTT assay. Data are expressed as percentage of control (100%), means + SEM, n = 3–5. *p < 0.05; **p < 0.005; ***p < 0.001 *vs*. vehicle control (paired t-test).

We next investigated the impact of diflapolin on other enzymes within the AA cascade that are involved in the biosynthesis of various eicosanoids in addition to FLAP and sEH. Besides FLAP, the LTC_4_S and mPGES-1 belong to the membrane-associated proteins in eicosanoid and glutathione metabolism (MAPEG) family sharing high sequence and structure homology to FLAP^33, 34^. Potent FLAP inhibitors (like MK886 or BRP-187) inhibit the MAPEG family members LTC_4_S and mPGES-1 as well^27, 35, 36^. In contrast, diflapolin failed to inhibit LTC_4_S and mPGES-1 activity in cell-free assays up to 10 µM (Fig. 3a,b), which indicates a high target specificity of diflapolin among the MAPEGs. Also, diflapolin did not significantly inhibit the activities of COX-1 and -2 in cell-free assays (Fig. 3c), whereas the reference drug indomethacin blocked COX activities, as expected. The activities of other LOXs (i.e. 12-LOX and 15-LOX) in neutrophils incubated with A23187 and 20 µM AA were not inhibited by diflapolin. In contrast, 15-HETE formation was concentration-dependently elevated (up to 200% of the vehicle control) (Fig. 3d). Since diflapolin was most potent in leukocytes to suppress 5-LOX product formation from endogenous AA but ineffective when high concentrations of exogenous AA were supplied, the compound could act at the level of AA release. However, in [^3^H] AA-pre-labelled neutrophils, diflapolin even at high concentrations (1 µM) did not inhibit the release of AA upon A23187-stimulation, as compared to the cPLA_2_ inhibitor RSC-3388 that suppressed AA liberation (Fig. 3e). Finally, detrimental effects on cellular viability could be excluded, as diflapolin (10 µM) did not affect the viability of monocytes in a MTT assay at 24 or 48 hrs, while staurosporine (3 µM, positive control) strongly impaired cell viability under these conditions (Fig. 3f). Taken together, diflapolin dually and strongly inhibits FLAP and sEH with target specificity as it did not interfere with other AA pathway-related enzymes (LTA_4_-H, COX-1/2, cPLA_2_, 12/15-LOXs) and FLAP-related MAPEG enzymes such as mPGES_1_ and LTC_4_S.

### Effects of diflapolin on 5-LOX subcellular redistribution and 5-LOX/FLAP complex assembly

**Figure 4:**
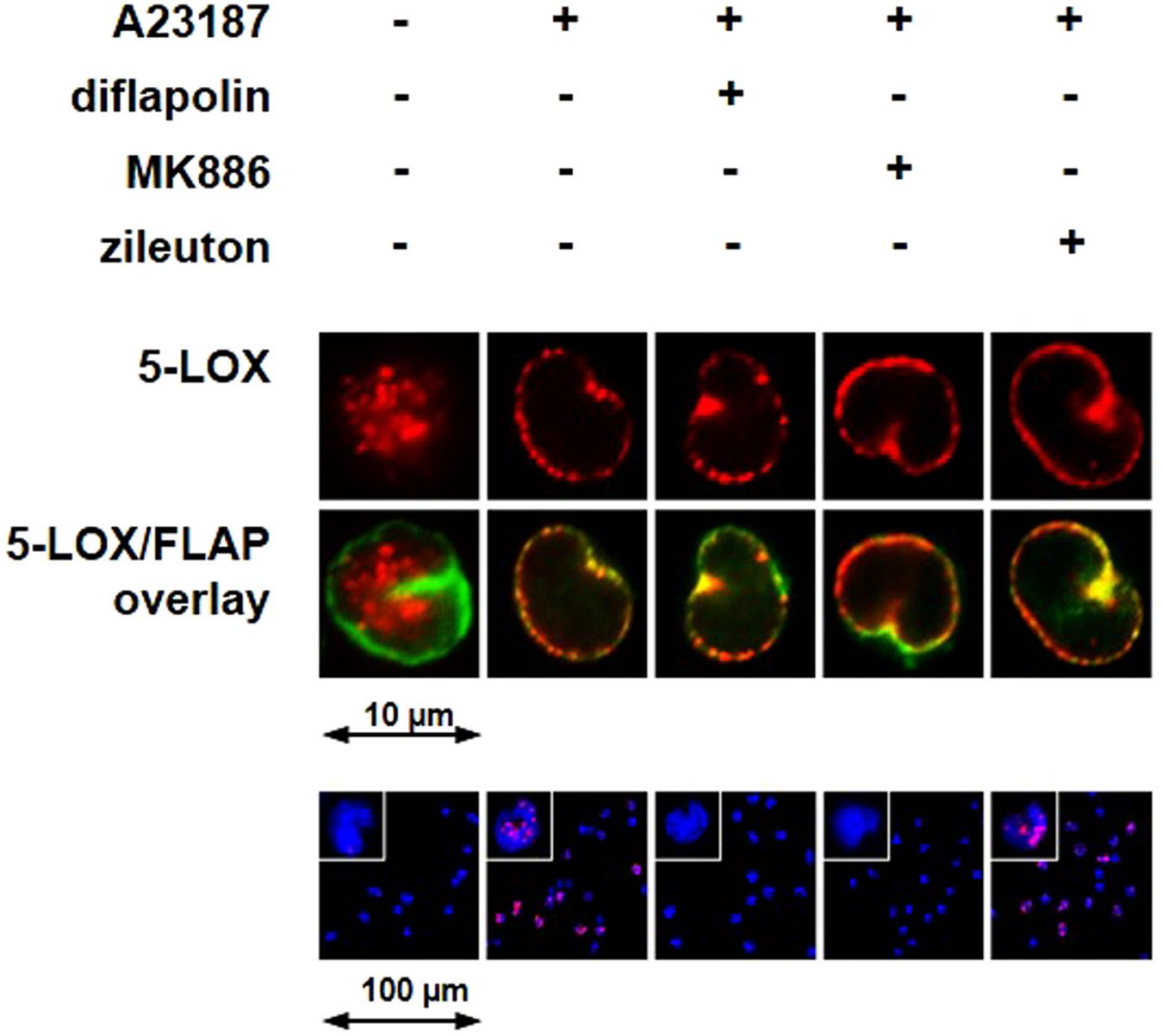
Effect of diflapolin on 5-LOX subcellular redistribution and 5-LOX/FLAP interaction. Cells were pretreated with diflapolin (1 µM), MK886 (0.3 µM), zileuton (3 µM) or 0.1% DMSO for 15 min, and then stimulated with 2.5 µM Ca^2+^-ionophore A23187 for 10 min at 37 °C. Top panel: immunofluorescence microscopy was used to determine 5-LOX subcellular localization. Single images (top lane) show 5-LOX staining (red) and the overlay of 5-LOX (red) and FLAP (green) (middle lane). Results are representative for 100 individual cells of three independent experiments. Lower panel: *in situ* PLA was applied to determine 5-LOX/FLAP complex assembly in monocytes (lower panel) using antibodies against 5-LOX and FLAP. DAPI stains the nucleus (blue), and PLA signals by 5-LOX/FLAP complexes are stained in magenta. Results are representative for 100 individual cells of three independent experiments.

5-LOX, a soluble cytosolic or intranuclear enzyme in resting leukocytes, translocates to the nuclear envelope upon cell activation and co-localizes with FLAP at the nuclear membrane to form a tight LT biosynthetic complex^37, 38^. Immunofluorescence microscopy studies using human neutrophils or monocytes showed that neither diflapolin nor the FLAP inhibitor MK886 or the 5-LOX inhibitor zileuton prevented co-localization of 5-LOX with FLAP (Fig. 4). However, diflapolin efficiently prevented the tight 5-LOX/FLAP complex assembly, visualized by proximity-ligation assay (PLA) (Fig. 4), a common feature of FLAP inhibitors^37^. MK886 gave comparable effects, whereas the 5-LOX inhibitor zileuton failed in this respect.

### Diflapolin exhibits potent anti-inflammatory properties in *in*-*vivo* experiments

**Figure 5:**
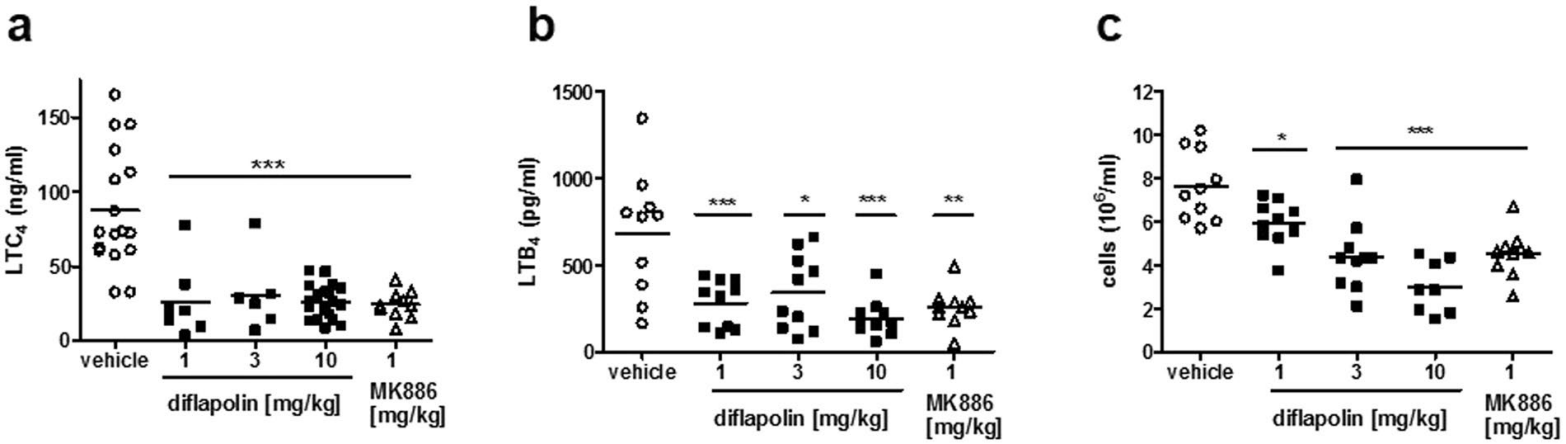
Effect of diflapolin on LTC_4_ and LTB_4_ formation, and cell recruitment in zymosan-induced peritonitis. Male mice (n = 6–19, each group) were treated i.p. with diflapolin, MK886 or vehicle, 30 min before before induction of zymosan-induced peritonitis. (**a**) LTC_4_ levels were analysed 30 min after zymosan injection by ELISA. (**b**) LTB_4_ formation and (**c**) cell infiltration were analysed 4 h after zymosan injection. *p < 0.05; **p < 0.01, ***p < 0.001 versus vehicle (Anova + Bonferroni).

We next investigated the anti-inflammatory effectiveness of diflapolin in the zymosan-induced peritonitis mouse model^39^ that is strongly related to the pathophysiological activities of LTs. Diflapolin pre-treatment (1, 3 and 10 mg/kg, i.p. 30 min before zymosan injection) induced a significant reduction of LTC_4_ and LTB_4_ peritoneal levels, starting from the dose of 1 mg/kg (Fig. 5a and b) and comparable to the effect of MK886 (1 mg/kg, i.p. 30 min before zymosan). Since LTB_4_ is a major chemoattractant for leukocytes, diflapolin and MK886 caused concomitant block of leukocyte recruitment, which was dose-dependent for diflapolin (Fig. 5c). Accordingly, at the dose of 10 mg/kg, diflapolin inhibited the activity of MPO, a typical marker protein for neutrophils to 52.8 ± 12.2% (mean ± SEM) *vs*. vehicle control and reduced vascular permeability to 55.7 ± 14.4% (mean ± SEM) *vs*. vehicle control (Table 1), compared to inhibitory effects of MK886 (1 and 3 mg/kg) to 58.5 ± 10.6% and 48.6 ± 3.2%, respectively.

**Table 1:** Effect of diflapolin on vascular permeability and myeloperoxidase (MPO) activity in zymosan-induced peritonitis.

	vascular permeability		myeloperoxidase activity	
	absorption (610 nm)	% of vehicle control	U/mL	% of vehicle control
vehicle	0.623 ± 0.04	100	1.23 ± 0.13	100
diflapolin 10 mg/kg	0.347 ± 0.09	55.7 ± 14.4	0.65 ± 0.15	52.8 ± 12.2
MK886 1 mg/kg	n.d.	n.d.	0.72 ± 0.13	58.5 ± 10.6
MK886 3 mg/kg	0.303 ± 0.02	48.6 ± 3.2	n.d.	n.d.

Male mice (n = 5 for vascular permeability, n = 7 for MPO; each group) were treated i.p. with diflapolin, MK886, or vehicle, 30 min before induction of zymosan-induced peritonitis. Analysis of vascular permeability and MPO was performed 30 min and 4 h, respectively, after zymosan injection. Data are given means ± S.E.M, (vascular permeability n = 5; myeloperoxidase activity n = 7) *p < 0.05 versus vehicle (Anova + Bonferroni). n.d., not determined.

## Discussion

Our recent pharmacophore-based virtual screening campaign for dual FLAP/sEH inhibitors proposed diflapolin as most promising hit and novel chemotype targeting both FLAP and sEH^15^. Here, we disclose diflapolin as a potent, dual inhibitor of FLAP and sEH with marked anti-inflammatory efficacy *in vivo* and high target selectivity. Side-by-side studies of diflapolin with the “FLAP benchmark inhibitor” MK886^40^ revealed comparable potencies for inhibition of 5-LOX product biosynthesis in human leukocytes *in vitro*, and about equal effectiveness in suppression of LT formation and inflammatory properties *in vivo* using murine zymosan-induced peritonitis models.

The identification of novel chemotypes as FLAP inhibitors is hampered due to the lack of distinct assays that unequivocally proof direct and functional interference of a given compound with FLAP^23^. Thus far, no enzymatic activity has been assigned to FLAP that can be exploited as read-out in FLAP inhibitor discovery approaches. Moreover, FLAP does not support 5-LOX activity in cell-free assays (e.g. homogenates)^41^. Nevertheless, FLAP is essential for LT biosynthesis in intact cells and *in vivo*, as reflected by results from various pharmacological approaches^40^ and from gene intervention using FLAP knock-out mice^42^. Experimental evidence suggests that FLAP operates as a 5-LOX helper protein for transforming AA to 5-HPETE and for dehydration of 5-HPETE to LTA_4_^29, 43^. FLAP is able to bind AA^26^ and to stimulate conversion of AA and 5-HPETE by 5-LOX^44^, and along these lines AA or 5-HPETE were required for *in situ* 5-LOX/FLAP complex assembly at the nuclear membrane in activated cells^37, 38^. Together, it appears that FLAP binds released AA and/or *de novo*-formed 5-HPETE and transfers them to 5-LOX, thus permitting optimal access of 5-LOX towards its substrates.

In light of these facts, assignment of a small molecule as FLAP inhibitor requires certain characteristics, which are all fulfilled by diflapolin. First of all, diflapolin did not randomly emerge as 5-LOX product biosynthesis inhibitor but was identified in a target-directed screening campaign for dual FLAP/sEH inhibitors applying two independently created ligand-based pharmacophore models^15^. Second, diflapolin potently inhibited LT biosynthesis only in intact leukocytes (without being cytotoxic or suppressing AA substrate release) but did not directly affect the activity of isolated human recombinant 5-LOX or 5-LOX in leukocyte homogenates. Third, ample supply of exogenous AA strongly reduced the potency of diflapolin in stimulated neutrophils and monocytes, compatible with the proposed competition between AA and FLAP inhibitors for binding to FLAP^43^. Fourth, diflapolin prevented the agonist-induced 5-LOX/FLAP complex assembly at the nuclear membrane in monocytes, visualized by PLA^27, 37^, without blocking 5-LOX translocation to the nucleus. Finally, the most striking proof for diflapolin inhibiting 5-LOX product formation by acting on FLAP but not on 5-LOX, is deduced from the fact that diflapolin suppresses cellular 5-LOX activity in 5-LOX-transfected HEK293 cells only when FLAP was co-expressed, while in cells devoid of FLAP, diflapolin failed in this respect. Note that these features of diflapolin were shared also with the FLAP inhibitor MK886 but not with the direct 5-LOX inhibitor zileuton (refs 27, 29, 37, 41 and 45 and this study). In conclusion, diflapolin is a potent LT biosynthesis inhibitor that confers its activity via inhibition of FLAP.

Besides FLAP, diflapolin was identified as potential hit using pharmacophore models for sEH inhibitors^15^, and it potently inhibited the epoxide hydrolase activity of sEH in a cell-free assay (IC_50_ 20 nM), while the phosphatase activity was not affected. In contrast, the epoxide hydrolase activity of LTA_4_-H was not affected by diflapolin, indicating specificity for sEH. Interference of diflapolin with sEH is not surprising since the urea moiety is a typical structural feature of sEH inhibitors that prevent the degradation of EETs^12, 46^. EETs formed from AA by CYP enzymes display anti-inflammatory and antihypertensive properties, maintain vascular homeostasis, and act generally cardio-protective^20, 47^. Among several EET degrading pathways, sEH metabolizes EETs to the corresponding dihydroxyeicosatrienoic acids (DiHETrE) with accompanied loss of health-promoting benefits^20, 48^. Accordingly, inhibition of sEH elevates EET levels leading to various beneficial effects. Little is known about the physiological role of the phosphatase activity, but an influence on the regulation of the endothelial nitric oxide synthase (eNOS) and NO-mediated effect on endothelial cells was suggested^49^.

Because FLAP is a member of the MAPEG family, other structurally-related MAPEG members such as mPGES-1 and LTC_4_S might be targeted by diflapolin as well, which is the case for the FLAP inhibitors MK866^36, 50^ and BRP-187^27^ that interfere with all three of these proteins. Diflapolin had no impact on mPGES-1 and LTC_4_S activity up to 10 µM, suggesting target specificity within the MAPEG family. Moreover, other enzymes within the AA cascade including COX-1/2, epoxide hydrolase activity of LTA_4_-H, 12-LOX and 15-LOXs were not inhibited by diflapolin. Interestingly, formation of 15-HETE in neutrophil incubations was concentration-dependently increased by diflapolin, which might be explained by AA shunting towards the 15-LOX pathway. This may promote the formation of anti-inflammatory and pro-resolving LMs such as lipoxins, resolvins and protectins^51^. A shift from biosynthesis of pro-inflammatory eicosanoids and other oxylipins towards anti-inflammatory and pro-resolving LMs would certainly strengthen the power of dual FLAP/sEH inhibitors. Additionally, even though comprehensive data are yet not available, it is reasonable to assume that epoxy-fatty acids derived from other polyunsaturated fatty acids confer anti-inflammatory properties.

The current development of LT biosynthesis inhibitors as therapeutics focusses on FLAP inhibitors^23^. Early representatives such as the indole MK886^18^ and the quinoline BAY X-1005^19^ are highly effective *in vitro* but probably due to their high lipophilicity they suffered from strong plasma protein binding, competition with fatty acids and, as a consequence reduced activity *in vivo* ^17, 40^. However, more advanced compounds including the MK886 follow-up GSK2190915, the tetrahydrofuran derivative AZD6642 and the oxadiazole-based BI665915 are less prone to plasma protein binding with advantageous pharmacokinetics^23^. These compounds are under active development (some entered clinical trials), and they appear to have lower risks of side effects as compared to 5-LOX inhibitors^40^. Diflapolin is structurally unrelated to these above-mentioned chemotypes and represents the first FLAP inhibitor with a polar urea moiety (seemingly the pharmacophore for sEH interference) and high efficiency *in vivo*.

In order to evaluate the *in vivo* efficacy and anti-inflammatory potential of diflapolin, we utilized the zymosan-induced peritonitis mouse model that is well established as test system for studying LT biosynthesis *in vivo* ^39^. Our data show that diflapolin is about equally effective as MK886 in reducing LTB_4_ and LTC_4_ levels in the peritoneal exudates with consequent biological functions. LTB_4_ is a potent chemotactic agent for neutrophils^52^ and in fact, neutrophil infiltration into the peritoneal cavity was strongly reduced by diflapolin. Cys-LTs mediate plasma extravasation^53^ and diflapolin significantly impaired vascular permeability during peritonitis. Conclusively, diflapolin potently inhibits LT formation *in vivo* connected with anti-inflammatory activity.

We speculate that dual inhibition of FLAP and sEH inhibitor may have synergistic anti-inflammatory and cardio-protective actions. Indeed, increased levels of EETs seem to be cardio-protective^47^ and FLAP was reported to be linked to certain cardiovascular diseases^54^. DMLs that block sEH and COX or sEH and 5-LOX are proposed to have improved anti-inflammatory activities over compounds that interfere with only one target enzyme^12, 13, 21, 55^. Such DMLs dually targeting sEH and FLAP are thus far unknown. Future studies addressing the pharmacological relevance of suppression of LTs with accompanied elevation of EETs may reveal potential benefit in the therapy of inflammatory and cardiovascular diseases.

Taken together, here we provide substantial evidence that diflapolin acts as potent dual FLAP/sEH inhibitor with high target specificity. The compound lacks acute cytotoxicity and efficiently suppresses LT biosynthesis *in vivo* connected with potent anti-inflammatory activity in a mouse model. Based on these features, diflapolin might be a valuable chemical tool for studying the biology of FLAP and sEH, particularly as synergizing targets, and may represent a useful lead for evaluation of the therapeutic potential of dual FLAP and sEH inhibition in inflammatory and cardiovascular disorders.

## Materials and Methods

### Materials

Diflapolin was obtained from Specs designated AQ-090/41740539 (Zoetermeer, Netherlands). Bovine serum albumin (BSA), EDTA, glutathione, saccharose, and Tris, AppliChem (Darmstadt, Germany); L-glutamine, BioChem GmbH (Karlsruhe, Germany); β-PGE_2_ and tritium-labeled [5,6,8,9,11,12,14,15-^3^H] labeled AA, Biotrend Chemicals GmbH (Köln, Germany); AA, PGB_1_, 3-phenyl-cyano(6-methoxy-2-naphthalenyl)methyl ester-2-oxiraneacetic acid (PHOME), LTC_4_-d5-methyl ester, LTA_4_ methyl ester, COX isoenzymes, and MK886, Cayman Chemical (Biomol, Hamburg, Germany); acetonitrile, Dulbecco’s modified Eagle’s high glucose medium with glutamine, geneticin, penicillin/streptomycin-solution and trypsin-EDTA, GE Healthcare Life Science (Freiburg, Germany); PGH_2_, Larodan Fine Chemicals (Stockholm, SWE); Alexa Fluor 488 goat anti-rabbit, Alexa Fluor 555 goat anti-mouse, hygromycin B, Lipofectamine LTX Reagent Plus, and non-immune goat serum and Sf-900™ II SFM, Invitrogen (Darmstadt, Germany). DMSO, Merck (Darmstadt, Germany); IL-1β, ReproTech (Hamburg, Germany); ATP, Roche (Mannheim, Germany); SDS, Roth GmbH (Karlsruhe, Germany); zileuton, Sequoia Research Products (Oxford, UK); Dulbecco’s Buffer Substance (PBS), SERVA Electrophoresis (Heidelberg, Germany); dithiothreitol and HPLC solvents, VWR (Darmstadt, Germany); Ca^2+^-ionophore A23187, dextrane, fetal calf serum (FCS), 3-(4,5-dimethylthiazol-2-yl)-2,5-diphenyltetrazolium bromide (MTT), non-essential amino acids, RPMI 1640 Medium, phenylmethanesulfonyl fluoride, soybean trypsin inhibitor, lysozyme, leupeptin, fatty acid free BSA, Duolink detection reagents red, Duolink PLA probe anti-rabbit PLUS, Duolink PLA probe anti-mouse MINUS, ATP agarose, Duolink wash buffers as well as other chemicals were from Sigma-Aldrich (Taufkirchen, Germany).

### Cell isolation and cell culture

Peripheral blood (University Hospital Jena, Germany) was collected from fasted healthy adult donors that were informed about the aim of the study and gave written consent. The protocols for experiments with neutrophils or monocytes were approved by the ethical commission of the Friedrich-Schiller-University Jena (approval number 4292-12/14). All methods were performed in accordance with the relevant guidelines and regulations. Leukocyte concentrates were obtained by centrifugation (4000 × g, 20 min, 20 °C) of heparinized blood preparations. Neutrophils and monocytes were immediately isolated as described before^56^. In brief, leukocyte concentrates were subjected to dextran sedimentation and centrifuged on lymphocyte separation medium (LSM 1077, PAA, Coelbe, Germany). For isolation of pelleted neutrophils, remaining erythrocytes were removed by hypotonic lysis, washed twice with ice-cold PBS and resuspended in medium to a cell density described for the respective experiments. Monocytes were separated from peripheral blood mononuclear cells (PBMC) by adherence to cell culture flasks (Greiner Bio-one, Nuertingen, Germany) for 1.5 h (37 °C, 5% CO_2_) in RPMI 1640 containing L-glutamine (1 mM), heat-inactivated FCS (10%), penicillin (100 U/mL) and streptomycin (100 µg/mL), followed by cell-scraping and resuspension in PBS.

HEK293 cells were cultured in monolayers (37 °C, 5% CO_2_) in DMEM containing FCS (10%), penicillin (100 U/mL) and streptomycin (100 µg/mL). HEK293 cell lines stably expressing 5-LOX with or without FLAP were selected using geneticin (400 µg/mL) with or without hygromycin (200 µg/mL), respectively, as previously described^29^. Transfection of HEK293 was performed using pcDNA3.1 plasmids and lipofectamine according to the manufacturer’s instructions (Invitrogen, Darmstadt, Germany).

Sf9 cells were cultured in monolayers at 27 °C in Sf-900 II SFM medium containing FCS (10%), penicillin (100 U/mL) and streptomycin (100 µg/mL).

HepG2 cells were cultured as monolayers in RPMI 1640 containing FCS (10%), penicillin (100 U/mL) and streptomycin (100 µg/mL) at 37 °C, 5% CO_2_.

### Expression, purification and activity assay of human recombinant 5-LOX

Human recombinant 5-LOX was expressed in *E*.*coli* BL21 transformed with pT3-5-LOX plasmid at 30 °C overnight as described before^57^. Cells were lysed in lysis buffer containing triethanolamine (50 mM, pH 8.0), EDTA (5 mM), phenylmethanesulfonyl fluoride (1 mM), soybean trypsin inhibitor (60 µg/mL), dithiothreitol (2 mM) and lysozyme (1 mg/mL) and homogenized by sonification (3 × 15 s). 5-LOX was purified from the 40,000 × g supernatant (20 min, 4 °C) using an ATP-agarose column and diluted with PBS buffer containing 1 mM EDTA. To determine 5-LOX product formation, aliquots (0.5 µg purified 5-LOX in 1 mL PBS plus 1 mM EDTA) were pre-incubated with the test compounds or vehicle (0.1% DMSO) on ice for 15 min and then stimulated with 20 µM AA and CaCl_2_ (2 mM) for 10 min at 37 °C. The reaction was stopped with one volume of ice-cold methanol and 5-LOX products were analyzed by RP-HPLC as previously described^58^. Briefly, 530 µl acidified PBS and 200 ng of internal PGB_1_ standard were added and solid phase extraction using C18 RP-columns (100 mg, UCT, Bristol, PA, USA) was performed. After elution with methanol, samples were analyzed by RP-HPLC using a C-18 Radial-PAK column (Waters, Eschborn, Germany). Unless stated otherwise, 5-LOX products include all-trans-isomers of LTB_4_ and 5-HpETE as well as its corresponding alcohol 5-HETE.

### Determination of 5-LOX products in intact cells and corresponding homogenates

In order to examine 5-LOX product formation in intact human neutrophils and monocytes, freshly isolated cells were resuspended in PBS buffer containing 0.1% glucose and 1 mM CaCl_2_ (PGC buffer) to a final cell density of 5 × 10^6^ or 2 × 10^6^, respectively. Cells were pre-incubated with the test compounds or vehicle (0.1% DMSO) at 37 °C for 15 min prior to stimulation with 2.5 µM Ca^2+^-ionophore A23187 for 10 min (37 °C) with or without supplementation of the indicated concentrations of AA. 5-LOX product formation was stopped by addition of one volume of ice-cold methanol, samples were subjected to solid phase extraction after addition of 200 ng PGB_1_ as internal standard and 5-LOX products were analyzed by RP-HPLC as described above.

Determination of 5-LOX products in corresponding homogenates was performed by resuspending neutrophils (final density of 5 × 10^6^ cells/mL) or monocytes (2 × 10^6^ cells/mL) in PBS containing 1 mM EDTA and sonicated on ice (3 × 15 s). Aliquots of homogenates were pre-incubated with the test compounds or vehicle (0.1% DMSO) on ice for 15 min and stimulated with 20 µM AA and CaCl_2_ (2 mM) at 37 °C for 10 min. 5-LOX product formation was assayed as described for intact cells above.

For analysis of 5-LOX product formation in HEK293 cells stably expressing 5-LOX with or without FLAP, cells were harvested by trypsinization, pelleted (1,200 rpm, 5 min, 4 °C) and resuspended in PGC buffer to a final concentration of 1 × 10^6^ cells/mL. Aliquots were pre-incubated with test compounds or 0.1% DMSO for 15 min, respectively, and stimulated with 2.5 µM A23187 and 3 µM AA. After termination of the incubations by addition of one volume of ice-cold methanol, samples were subjected to solid phase extraction and analysis of 5-LOX products as described above.

### Determination of LTA_4_ hydrolase activity

In order to determine the activity of LTA_4_-H, aliquots of human recombinant 5-LOX (0.5 µg) and 10 µg of human recombinant LTA_4_-H (kindly provided by Dr. E. Proschak, Goethe University, Frankfurt, Germany) were suspended in 1 mL PBS containing 1 mM EDTA and pre-incubated with test compounds or vehicle (0.1% DMSO) for 10 min on ice. The specific LTA_4_-H inhibitor SC57461A (0.3 µM) was used as reference drug. Subsequently, incubations were stimulated with 20 µM AA and 2 mM CaCl_2_ for additional 10 min at 37 °C. The reaction was stopped by 1 volume ice-cold methanol, 530 µL acidified PBS and 200 ng of PGB_1_ as internal standard were added and subjected to solid phase extraction. All LTB_4_ isomers were analyzed by HPLC as described above.

### Expression, purification and activity assays of human recombinant sEH

Human recombinant sEH was expressed and purified as reported before^59^. In brief, Sf9 cells were infected with a recombinant baculovirus (kindly provided by Dr. B. Hammock, University of California, Davis, CA). 72 hrs post-transfection, cells were pelleted and sonicated (3 × 10 sec at 4 °C) in lysis buffer containing NaHPO_4_ (50 mM, pH 8), NaCl (300 mM), glycerol (10%), EDTA (1 mM), phenylmethanesulfonyl fluoride (1 mM), leupeptin (10 µg/mL), and soybean trypsin inhibitor (60 µg/mL). Supernatants after centrifugation at 100,000 × g (60 min, 4 °C) were subjected to a benzylthio-sepharose-affinity chromatography in order to purify sEH by elution with 4-fluorochalcone oxide in PBS containing DTT (1 mM) and EDTA (1 mM). Dialyzed and concentrated (Millipore Amicon-Ultra-15 centrifugal filter) enzyme solution was assayed for total protein with Bio-Rad protein detection kit (Bio-Rad Laboratories, Munich, Germany) and the epoxide hydrolase activity was determined by using a fluorescence-based assay as described before^60^. Briefly, sEH was diluted in Tris buffer (25 mM, pH 7) supplemented with BSA (0.1 mg/mL) to an appropriate enzyme concentration and pre-incubated with test compounds or vehicle (0.1% DMSO) for 10 min at room temperature (RT). The reaction was started by addition of 50 µM 3-phenyl-cyano(6-methoxy-2-naphthalenyl)methyl ester-2-oxiraneacetic acid (PHOME), a non-fluorescent compound that is enzymatically converted into fluorescent 6-methoxy-naphtaldehyde at RT. After 60 min, reactions were stopped by ZnSO_4_ (200 mM) and fluorescence was detected (λ_em_ 465 nm, λ_ex_ 330 nm) and potential fluorescence of test compounds was subtracted from the read-out, if required.

In order to analyze the phosphatase activity of sEH, a recently published assay^61^ was performed. In brief, purified sEH-phosphatase-domain was pre-incubated with test compounds or vehicle (0.1% DMSO), in acetate buffer (50 mM, pH 5.8) containing MgCl_2_ (10 mM) and Triton-X-100 (0.01%) for 30 min at RT prior to addition of 6,8-difluoro-4-methylumbelliferyl phosphate (DiFMUP, 300 µM). Phosphatase activity was assayed by measurement of fluorescence (λ_ex_ 360 nm, λ_em_ 450 nm) of the dephosphorylated DiFMU for 45 min at 37 °C.

### Determination of 14,15-DiHETrE-formation in HepG2 cells and in a cell-free assay

To assay the activity of sEH in a cell-based model, HepG2 cells were harvested by trypsinization, pelleted (1,200 rpm, 5 min, 4 °C) and resuspended in PGC buffer to a final concentration of 1.5 × 10^6^ cells/mL. Cells were pre-incubated with test compounds or 0.1% DMSO at 37 °C for 15 min, and incubated with 1.5 µM 14,15-EET (Cayman Chemical, Biomol, Hamburg, Germany) for 30 min at 37 °C. After termination of the reactions with one volume of ice-cold methanol, 1.31 ng d8-5(S)-HETE and 1.36 ng d4-LTB_4_ (Cayman Chemical, Biomol, Hamburg, Germany) were added as internal standards and samples were subjected to solid phase extraction. Briefly, samples were acidified with four volumes of MilliQ water pH 3.5 containing PBS-HCl and subjected to Waters Sep-Pak® Vac 6cc columns (Waters, Milford, MA, USA), washed once with MilliQ and hexane, and eluted with methylformiate. The nitrogen-dried samples were dissolved in 50% methanol, and 14,15-DiHETrE formation was measured by UPLC-MS/MS using an Acquity™ UPLC system (Waters, Milford, MA, USA) and a QTRAP 5500 Mass Spectrometer (ABSciex, Darmstadt, Germany) equipped with a Turbo V™ Source and electrospray ionization (ESI). In brief, lipid mediators were separated using a Sep-Pak C18 35 cc Vac Cartridge, 10 g Sorbent per Cartridge, 55–105 µm Particle Size, 10/pk (Waters, Milford, MA, USA) at 50 °C with a flow rate of 0.3 mL/min. MilliQ water (A) and methanol (B) both acidified with 0.1% acetic acid were used as solvents with an increasing percentage of B starting at 42% and ending with 86% at 12.5 min followed by isocratic elution at 98% B for another 3 min. Analytes were detected by multiple reaction monitoring in the negative ion mode using the following transitions: 14,15-EET (m/z 319 → 219), 14,15-DiHETrE (m/z 337 → 207), d8-5(S)-HETE (m/z 327 → 116) and d4-LTB_4_ (m/z 339 → 197). Ion spray voltage was set to 4000 V, the heater temperature to 500 °C, the declustering potential to 50–80 eV, the entrance potential to 10 eV, the collision cell exit potential to 10–13 eV, collision energies of 10 eV, the spray gas pressure to 40 psi, medium collision gas and the curtain gas pressure to 35 psi.

In order to confirm the identity of detected metabolites, human recombinant sEH was diluted in 25 mM Tris buffer pH 7.0 to a final concentration of 0.3 µg/mL and pre-incubated with test compounds or 0.1% DMSO on ice for 15 min and stimulated with 14,15-EET (1.5 µM, 30 min, 37 °C). Extraction and detection of metabolites was performed as described for the cell-based assay.

### Determination of microsomal PGE_2_ synthase activity

To perform the analysis of microsomal PGE_2_ synthase (mPGES)-1 activity, microsomal preparations of A549 cells were obtained as described before^62^. Briefly, A549 cells were cultivated in DMEM medium containing FCS (2%) and IL-1β (2 ng/mL) for 72 h (37 °C, 5% CO_2_). Cells were harvested and resuspended in homogenization buffer consisting of potassium phosphate (0.1 M, pH 7.4), phenylmethanesulfonyl fluoride (1 mM), soybean trypsin inhibitor (60 µg/mL), leupeptin (1 µg/mL), glutathione (2.5 mM), and sucrose (250 mM). After freezing the cells in liquid nitrogen and sonication (3 × 20 s), a differential centrifugation at 10,000 g (10 min, 4 °C) and 174,000 × g (60 min, 4 °C) was performed and pellets were resuspended in homogenization buffer. To assay mPGES-1 activity, microsomes were diluted in potassium phosphate buffer (0.1 M, pH 7.4) with glutathione (2 mM) and pre-incubated with the test compounds or vehicle (0.1% DMSO) on ice for 15 min. After stimulation (1 min, 4 °C) with 20 µM PGH_2_ the reaction was terminated by addition of stop solution containing FeCl_3_ (40 mM), citric acid (80 mM), and 11β-PGE_2_ (10 µM as internal standard) and analyzed for PGE_2_ product formation by RP-HPLC as reported before^62^.

### Determination of LTC_4_ synthase (LTC_4_S) activity

LTC_4_S activity was assayed by using microsomes of HEK293 cells stably expressing LTC_4_S, as previously published^63^. In brief, HEK293 expressing LTC_4_S were cultivated as described above and selected using geneticin (400 µg/mL). Isolation of microsomes was performed as described for mPGES-1 above and microsomes were diluted in potassium phosphate buffer (0.1 M, pH 7.4) with glutathione (5 mM) to a final concentration of 2.5 µg protein per mL. After pre-incubation with the test compounds or vehicle (2% DMSO) for 10 min at 4 °C, reactions were started by addition of 1 µM LTA_4_-methyl ester and stopped by addition of one volume of ice-cold methanol after 10 min of incubation at 4 °C. To determine enzyme activity, acidified PBS and d_5_-LTC_4_-methyl ester (5 ng) as internal standard were added prior solid phase extraction and LTC_4_-methyl ester formation was analyzed by UPLC-MS/MS as described^63^.

### Determination of COX activity

COX activity was assayed by using purified ovine COX-1 and recombinant human COX-2, respectively. Enzymes were diluted in Tris buffer (100 mM, pH 8) supplemented with glutathione (5 mM), EDTA (100 µM) and hemoglobin (5 µM) to a final concentration of 50 U/mL (COX-1) or 20 U/mL (COX-2) and pre-incubated with test compounds or vehicle (0.1% DMSO) for 5 min at RT. After 30 sec at 37 °C, reactions were started with 5 µM AA (COX-1) or 2 µM AA (COX-2) and stopped after 5 min at 37 °C by addition of one volume of ice-cold methanol. Solid phase extraction was performed as described above after addition of 200 ng of internal PGB_1_ standard and COX product formation was determined by analysis of 12-HHT formation as reported before^64^.

### Analysis of acute cytotoxicity

Acute cytotoxicity of the compounds was determined using freshly isolated monocytes. Cells (0.2 × 10^6^) per sample were seeded in 100 µL buffer on 96-well plates and treated with the test compounds and appropriate controls for 24 or 48 hrs (37 °C, 5% CO_2_). After addition of MTT (5 mg/mL) for 2 h (37 °C, 5% CO_2_), cells were lysed by SDS treatment (10%, pH 4.5) for 16–20 hrs and formazan formation was determined by measurement of absorbance at 570 nm.

### Determination of [^3^H]-labeled arachidonic acid release

Freshly isolated human neutrophils were resuspended in RPMI 1640 medium to a final cell density of 10^7^ cells/mL and incubated with 0.5 µCi/mL of [^3^H]-labeled arachidonic acid ([^3^H]-AA), corresponding to a concentration of 5 nM of the fatty acid, for 2 hrs (37 °C, 5% CO_2_). Cells were washed twice to remove unincorporated [^3^H]-AA and resuspended in PBS containing glucose (0.1%), fatty acid-free BSA (2 mg/mL) and CaCl_2_ (1 mM). Aliquots of 10^7^ cells were pre-incubated with the test compounds or vehicle (0.1% DMSO) at 37 °C for 10 min and stimulated with 2.5 µM A23187 for another 10 min. The reaction was stopped on ice and cells were centrifuged at 1,200 rpm (10 min, 4 °C). The collected supernatants were combined with 2 mL of liquid scintillation counting solution (Rotiszint eco plus, Carl Roth, Karlsruhe, Germany) and assayed for radioactivity by scintillation counting (Micro Beta Trilux, Perkin Elmer, Waltham, MA).

### Immunofluorescence microscopy (IF) and proximity ligation assay (PLA)

In order to investigate cellular redistribution of 5-LOX and FLAP in monocytes, freshly isolated PBMC were seeded onto glass coverslips in RPMI 1640 containing L-glutamine (1 mM), heat-inactivated FCS (10%), penicillin (100 U/mL) and streptomycin (100 µg/mL) for 1.5 hrs (37 °C, 5% CO_2_). Cells were washed twice with PBS and pre-incubated with test compounds or vehicle control (0.1% DMSO) in PGC buffer for 15 min (37 °C) prior to stimulation with A23187 (2.5 µM, 10 min, 37 °C). The incubations were stopped by paraformaldehyde fixation (4%, 20 min, RT) and cells were permeabilized using 100% ice-cold acetone (5 min, 4 °C). Blocking with non-immune goat serum (30 min, RT) was performed prior to overnight incubation (4 °C) with primary monoclonal-mouse-anti-5-LOX antibody (1:100 dilution, a generous gift from Dr. D. Steinhilber, Goethe University Frankfurt, Germany) and polyclonal-rabbit-anti-FLAP antibody (5 µg/mL, Abcam, Cambridge, UK). Incubation with fluorophore-labeled secondary Alexa Fluor 488 goat anti-rabbit (1:1000) and Alexa Fluor 555 goat anti-mouse (1:1000) was processed for 30 min in the dark at RT and nuclear DNA was stained with DAPI-containing ProLong diamond antifade mountant (Invitrogen, Darmstadt, Germany) on glass slides. Cells were visualized by a Zeiss Axiovert 200 M microscope, and a Plan Neofluar × 100/1.30 Oil (DIC III) objective (Carl Zeiss, Jena, Germany) and image acquisition was performed using an AxioCam MR camera (Carl Zeiss).

*In situ* protein interaction of 5-LOX and FLAP was analyzed by proximity ligation assay as described before^37^ and referring to the manufacturer’s protocol^65^. In brief, freshly isolated monocytes were treated as described for IF above. Overnight incubations with primary antibodies were then treated for 1 h (37 °C) with oligonucleotide-labeled specific secondary antibodies (PLA probes anti-mouse MINUS and anti-rabbit PLUS). Formation of circled DNA sequences was induced by addition of ligase and oligonucleotide mixture (30 min at 37 °C). Rolling-circle-amplification of newly generated DNA template was performed (90 min, 37 °C) including hybridization of fluorescently-labeled oligonucleotides within the formed DNA strands, resulting in visualization of protein-protein interactions recognized as magenta-stained dots. Nuclear DNA staining with DAPI and image acquisition was performed as described above. Overview images were obtained using a Plan Neofluar 40/1.30 Oil (DIC III) objective (Carl Zeiss).

### Murine peritonitis model

The animal studies are reported in accordance with the ARRIVE guidelines for reporting animal research^66^. Male CD-1 mice (33–39 g, 8–9 weeks, Charles River Laboratories, Calco, Italy) were housed in a controlled environment (21 ± 2 °C) and provided with standard rodent chow and water ad libitum. Mice received a standard diet containing 5.7% fat, 18.9% protein and 57.3% carbohydrate (Global Diet 2018, ENVIGO, Italy). The fatty acid composition was according to Matias *et al*.^67^.

Prior to experiments, all mice were allowed to acclimate for 5 days and kept at 12 h light–dark schedule, in which experiments were performed during the light phase. Animal care was in compliance with Italian regulations on protection of animals used for experimental and other scientific purpose (Ministerial Decree 116/92) and with the European Economic Community regulations (Official Journal of E.C. L 358/1 12/18/1986). Animal studies were approved by the local ethical committee of the University of Naples Federico II on 27 February 2014 (approval number 2014/18760). Mice were treated with diflapolin (1, 3 or 10 mg/kg), MK886 (1 or 3 mg/kg), zileuton (10 mg/kg) or vehicle (0.9% saline solution containing 2% DMSO), received as intraperitoneal (i.p.) injection, 30 min prior induction of peritonitis according to well-recognized experimental design for studying LT synthesis inhibitors in acute inflammation^27^. Zymosan (Sigma, Milan, Italy) was prepared and injected i.p. as a final suspension (2 mg/mL) in 0.9% saline solution after boiling, centrifugation and sonication. Peritoneal lavage (3 mL of cold PBS) was performed after CO_2_-euthanasia at indicated time points, followed by 60 sec of gentle manual massage. Two mL of exudates were collected and infiltrated cells were determined using a Burker chamber and vital trypan blue staining. Pelleted samples (18,000 × g, 5 min, 4 °C) were frozen (−80 °C) and assayed for myeloperoxidase (MPO) activity (pellet) or LTC_4_ and LTB_4_ formation (supernatant), respectively.

MPO of neutrophils was examined as follows: pellets from exudates were resuspended in PBS (50 mM, pH 6) containing 0.5% hexadecyltrimethyl-ammonium bromide and sonicated, followed by 3 freeze-thawing cycles and a final sonication. Supernatants of centrifuged samples (18,000 × g, 30 min) were added to a 96-well plate and reactions were initiated by addition of PBS (50 mM, pH 6) containing o-dianisidine (0.167 mg/mL) and hydrogen peroxide (0.0005%). Absorbance was monitored in the kinetic mode (Biorad Imark microplate) and levels of MPO were determined using a calibration curve with human neutrophils as reference standard. MPO levels were expressed as units MPO per mouse. LTC_4_ and LTB_4_ formation within the supernatants were determined by EIA (Enzo Life Sciences International Inc., Lörrach, Germany) according to manufacturer’s protocol^39^.

Vascular permeability was assessed according to a previous report^27^. Briefly, 0.3 mL of 0.9% saline solution supplemented with Evans blue dye (40 mg/kg) was injected intravenously (i.v.) into the caudal vein followed by immediate peritonitis induction (using zymosan). After 30 min, peritoneal lavage exudates of CO_2_-euthanized were collected as described above. Absorbance of the centrifuged supernatants (3,000 × g, 5 min) was measured at 650 nm (Beckman Coulter DU730).

### Statistics

Results are presented as mean ± standard error of the mean out of *n* independent experiments, where *n* represents the number of performed experiments on different days or with different donors or the number of animals for *in vivo* studies. IC_50_ values were calculated from at least 5 different concentrations using a nonlinear regression interpolation of semi-logarithmic graphs in GraphPad Prism (GraphPad Software Inc., San Diego, CA). Statistical evaluation was performed by one-way ANOVA using GraphPad InStat (Graphpad Software Inc., San Diego, CA) followed by a Bonferroni post-hoc test for multiple or student t-test for single comparisons, respectively. P-values < 0.05 were considered as significant.
